# Clinical and sociodemographic determinants of Impulse Control Disorders in bipolar patients

**DOI:** 10.1192/j.eurpsy.2026.11080

**Published:** 2026-07-31

**Authors:** F. Sahnoun, S. Ellouze, R. Chakchouk, Y. Kammoun, N. Halouani, M. Turki, J. Aloulou

**Affiliations:** Psychiatry “B” Department, Hédi Chaker University Hospital, Sfax, Tunisia

## Abstract

**Introduction:**

Impulse Control Disorders (ICDs) often complicate the course of bipolar disorder, leading to poorer prognosis and treatment noncompliance. Understanding the clinical and sociodemographic determinants of ICDs could guide better identification and management of patients at risk.

**Objectives:**

To identify demographic, clinical, and therapeutic factors associated with the presence of ICDs among bipolar patients.

**Methods:**

This cross-sectional descriptive and analytical study included patients with bipolar disorder, during euthymia phase and receiving psychiatric follow-up in the Psychiatry “B” Department of Hédi Chaker University Hospital of Sfax, Tunisia. We collected sociodemographic, clinical, and treatment-related data. We used the modified Minnesota Impulsive Disorders Interview (mMIDI) to assess ICDs.

**Results:**

A total of 72 patients with bipolar disorder participated in our study. Their mean age was 44.13 years, and the sex ratio (F/H) was 1.88. The mean duration of illness was 17.04 years. Most patients (83.3%) had bipolar I disorder, and 77.8% had a history of hospitalization. Psychotic features were present in 72.2% of cases during the first affective episode. A history of suicide attempts was reported by 29.2% of patients. Overall, 33.3% of participants presented at least one impulse control disorder (ICD).

ICDs were more frequent among male patients (p = 0.049). A significant association was found between the presence of ICDs and psychotic features during the first affective episode (p = 0.041), a history of suicide attempts (p = 0.006), the number of suicide attempts (p = 0.014), and the number of hospitalizations (p=0.014). Conversely, the use of antidepressants during the illness appeared to have a protective effect against ICDs (p = 0.03).

**Conclusions:**

ICDs in bipolar disorder are associated with specific demographic and clinical characteristics, notably male sex, the presence of psychotic features during the first affective episode, and a history and higher number of suicide attempts and hospitalizations. In contrast, antidepressant treatment appeared to exert a protective effect. These findings underscore the importance of systematically screening for ICDs, particularly in patients presenting these risk factors.

**Disclosure of Interest:**

None Declared

